# The role of MAP2 kinases and p38 kinase in acute murine liver injury models

**DOI:** 10.1038/cddis.2017.295

**Published:** 2017-06-29

**Authors:** Jun Zhang, Robert W M Min, Khanh Le, Sheng Zhou, Mariam Aghajan, Tin A Than, Sanda Win, Neil Kaplowitz

**Affiliations:** 1 University of Southern California Research Center for Liver Disease, Keck School of Medicine, University of Southern California, Los Angeles, CA, USA; 2 IONIS Pharmaceuticals, Carlsbad, CA, USA

## Abstract

c-Jun N-terminal kinase (JNK) mediates hepatotoxicity through interaction of its phospho-activated form with a mitochondrial outer membrane protein, Sh3bp5 or Sab, leading to dephosphorylation of intermembrane Src and consequent impaired mitochondrial respiration and enhanced ROS release. ROS production from mitochondria activates MAP3 kinases, such as MLK3 and ASK1, which continue to activate a pathway to sustain JNK activation, and amplifies the toxic effect of acetaminophen (APAP) and TNF/galactosamine (TNF/GalN). Downstream of MAP3K, in various contexts MKK4 activates both JNK and p38 kinases and MKK7 activates only JNK. The relative role of MKK4 *versus* 7 in liver injury is largely unexplored, as is the potential role of p38 kinase, which might be a key mediator of toxicity in addition to JNK. Antisense oligonucleotides (ASO) to MKK4, MKK7 and p38 (*versus* scrambled control) were used for *in vivo* knockdown, and in some experiments PMH were used after *in vivo* knockdown. Mice were treated with APAP or TNF/GalN and injury assessed. MKK4 and MKK7 were expressed in liver and each was efficiently knocked down with two different ASOs. Massive liver injury and ALT elevation were abrogated by MKK4 but not MKK7 ASO pretreatment in both injury models. The protection was confirmed in PMH. Knockdown of MKK4 completely inhibited basal P-p38 in both cytoplasm and mitochondria. However, ALT levels and histologic injury in APAP-treated mice were not altered with p38 knockdown *versus* scrambled control. p38 knockdown significantly increased P-JNK levels in cytoplasm but not mitochondria after APAP treatment. In conclusion, MKK4 is the major MAP2K, which activates JNK in acute liver injury. p38, the other downstream target of MKK4, does not contribute to liver injury from APAP or TNF/galactosamine.

c-Jun N-terminal kinase (JNK) mediates hepatotoxicity through interaction of its phospho-activated form with a mitochondrial outer membrane protein,Sh3bp5 or Sab, leading to dephosphorylation of intermembrane Src and consequent impaired mitochondrial respiration and enhanced ROS release.^1, 2^ ROS release from mitochondria activates MAP3 kinases such as MLK3 and ASK1, which continue to activate a pathway to sustain JNK activation.^3, 4^ The JNK-mediated ROS production amplifies the toxic effect of acetaminophen (APAP) on mitochondria leading to mitochondrial permeability transition (MPT)-mediated necrosis.^5, 6, 7^ In the model of TNF/galactosamine (TNF/GalN)-induced hepatocyte apoptosis, the sustained JNK activation is critical in modulating the Bcl family members, which are the gatekeepers of mitochondrial outer membrane permeabilization leading to the release of cytochrome *c* and other apoptogenic proteins.^8, 9^

Downstream of ROS-responsive MAP3K, two dual-specificity MAP kinase kinases (MAP2K) are known to activate JNK. MKK4 activates both JNK and p38 kinases and MKK7 activates only JNK.^10, 11^ We have previously found that MKK4 is activated in APAP toxicity and translocates with JNK to mitochondria.^2^ However, the relative role of MKK4 *versus* 7 in liver injury is largely unexplored, as is the potential role of p38 kinase.

In the APAP model, many approaches to modulating MAP3K and JNK using small molecule inhibitors and genetic approaches have supported the role of JNK activation in liver injury. However, a recent study using liver-specific deletion of JNK1 in global JNK2 knockout mice has suggested the opposite, that is a protective role.^12^ Therefore, the current studies were conducted to gain further insight concerning this controversy by addressing the role of MAP2K to determine whether silencing the expression of either of the two MAP2K upstream of JNK would protect against acute liver injury. As we found that the MAP2K involved (MKK4) activates both JNK and p38, we also explored its possible role in liver injury.

## Results

**Figure 1:**
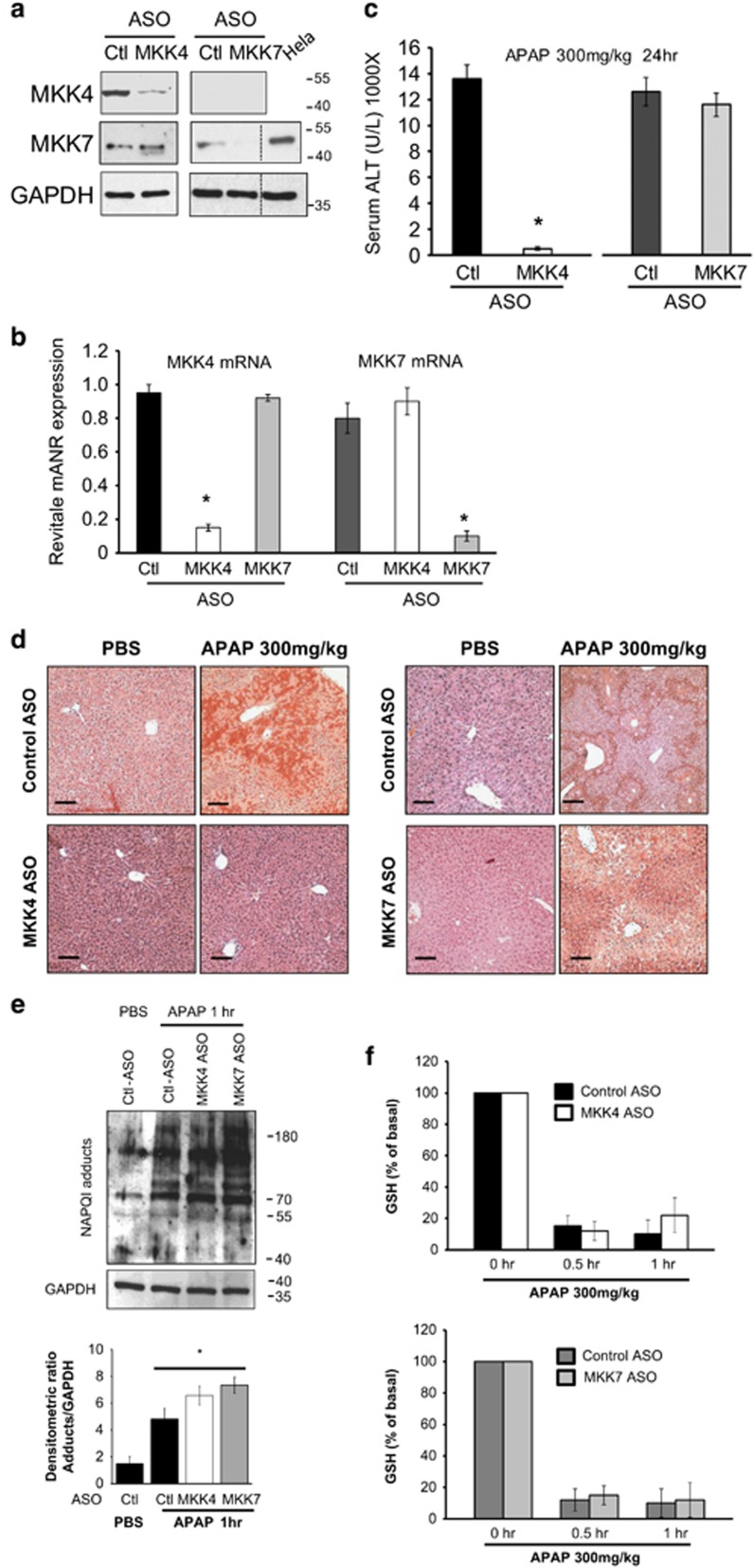
Comparison of knockdown of MKK4 *versus* MKK7 in acetaminophen induced necrotic liver injury model. After injection of control or MKK4 ASO or MKK7 ASO, the mice were treated with APAP (300 mg/kg) intraperitoneally. (**a**) Western blots of MKK4 and MKK7 knockdown in liver lysate. Hela cell lysate used as positive control. (**b**) Quantitative PCR of MKK4 or MKK7 in liver of control ASO *versus* MKK4 or MKK7 ASO-injected mice. MKK4 or MKK7 mRNA was normalized by housekeeping gene TBP (TATA box binding protein); *P<0.05; *n*=5 mice per each group. (**c**) Serum ALT 24 h after APAP. Mean±S.D., *n*=5 per group; *P<0.05 (*t*-test), Control *versus* MKK4 or MKK7 KD. (**d**) Representative hematoxylin and eosin-stained liver histology 24 h after APAP. Scale bars represent 100 *μ*m. (**e**) Effect of control, MKK4 ASO or MKK7 ASO on covalent binding of NAPQI. Western blot of liver homogenate was prepared 1 h after APAP (300 mg/kg) using anti-adduct antisera. (* indicates *versus* control PBS). (**f**) Liver GSH levels. Mean±S.D., *n*=5 per group and time point

To address the role of MKK4 or 7 in APAP toxicity, we first examined the basal level of expression and efficacy of knockdown of hepatic MKK4 or 7 *in vivo*. MKK4 and 7 expression in liver were efficiently knocked down by both their respective ASO: as shown by immunoblot and by qPCR. (>90%, Figures 1a and b). Neither ASO affected the other MKK indicating no cross reactivity. Knockdown of MKK4 markedly protected against histological liver necrosis and serum ALT elevation (Figures 1c and d) after a sublethal toxic dose of APAP (300 mg/kg). In contrast, MKK7 knockdown did not protect against liver injury. The MKK4 ASO treatment did not affect APAP metabolism (adducts and GSH depletion; Figures 1e and f).

**Figure 2:**
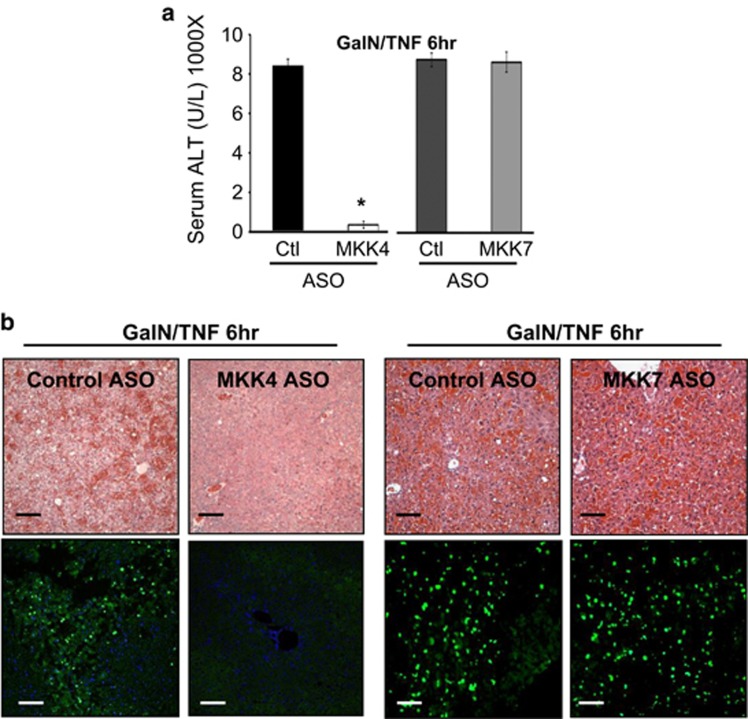
Effect of MKK4 knockdown in TNF/galactosamine apoptotic liver injury model. After injection of control, or MKK4 ASO, or MKK7 ASO, the mice were treated with TNF (12 *μ*g/kg)/GalN (800 mg/kg) intraperitoneally. (**a**) Serum ALT 6 h after TNF/GalN. Bars represent mean±S.D., *n*=5 per group; *P<0.05 (*t*-test), MKK4 KD *versus* control or MKK7 KD. (**b**) Comparison of hematoxylin and eosin or TUNEL stain of liver histology 6 h after TNF/GalN. Scale bars represent 100 *μ*m and quantitative microscopy is described in the 'Materials and Methods' section

The striking protection by MKK4 ASO, but not MKK7 ASO, was also seen in the TNF/galactosamine model of acute massive apoptosis, a model independent of drug metabolism and GSH detoxification. Massive hemorrhagic liver injury and ALT elevation were abrogated by MKK4 but not MKK7 ASO pretreatment (Figure 2). Furthermore, we used a second ASO targeting a different part of the mRNA sequences of MKK4 and MKK7 and observed similar efficient knockdown in the liver with the appropriate ASO. In both the APAP and TNF/GalN models, the second MKK4 ASO was also markedly protective, whereas the second MKK7 ASO was not protective (Supplementary Figures 1 and 2), as reflected in histological injury and serum ALT levels. These results support the conclusion that ASO knockdown of MKK4 is not protecting by an off-target effect of a single ASO.

**Figure 3:**
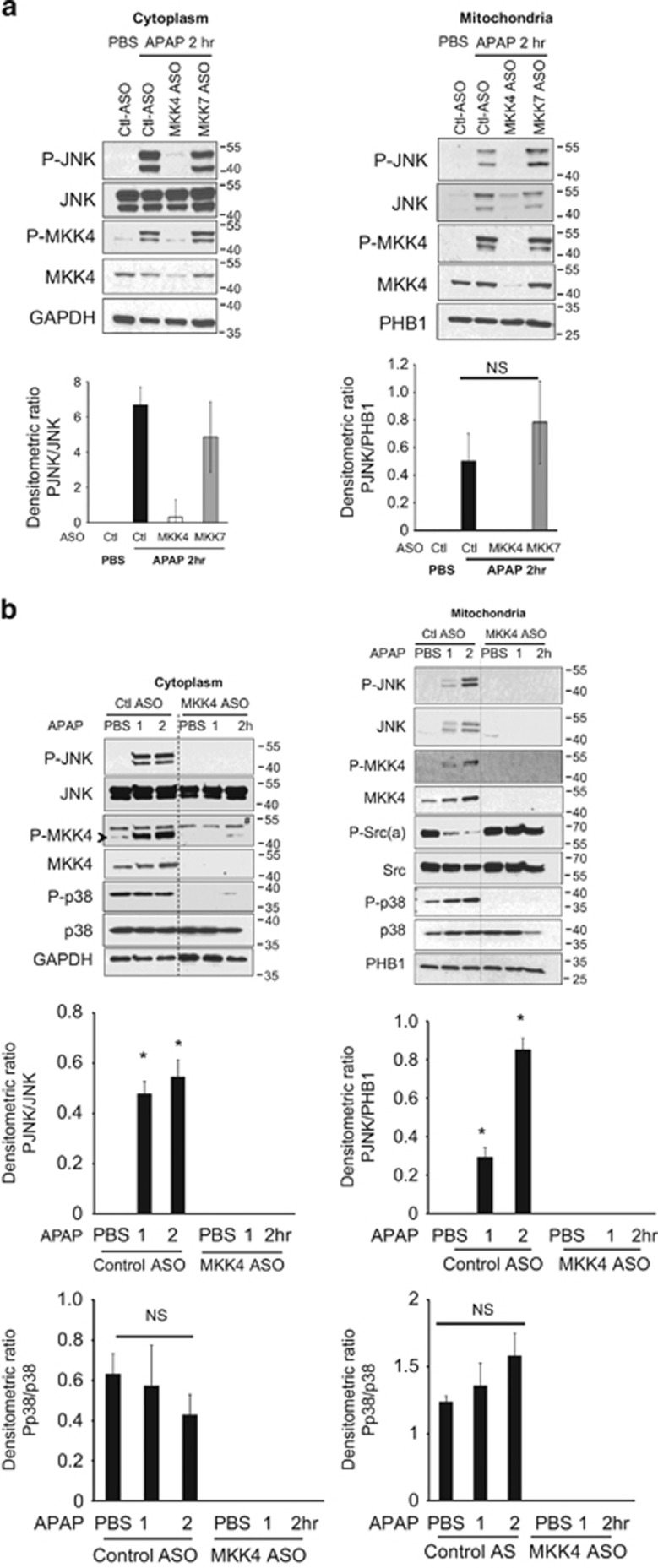
Effect of MKK4 or 7 knockdown on early signaling in cytoplasm and mitochondria in APAP toxicity. (**a**) Immunoblot analysis of JNK and MKK4 activation in cytoplasm and translocation to mitochondria 2 h after APAP (300 mg/kg) in ASO control *versus* MKK4 KD and MKK7 KD mice. (**b**) Immunoblots of cytoplasm and mitochondrial extracts at 1 and 2 h after APAP in control *versus* MKK4 KD livers. GAPDH (cytoplasm) and prohibitin (PHB1) (mitochondria) were loading controls. (* indicates *versus* control PBS; # indicates nonspecific band; NS, not significant). Bar graphs represent densitometric analysis of results from five separate mice per group. As JNK is not present in association with normal mitochondria, we used PHB1 for normalization of P-JNK in mitochondrial fraction

We examined the early signaling events in the liver before onset of APAP-induced hepatic necrosis. Consistent with protection from injury, the early activation of P-JNK and translocation to mitochondria was abrogated by MKK4 knockdown but not by MKK7 knockdown (Figure 3a). In addition the dephosphorylation of mitochondrial Src, which is critical in promoting ROS release and MAP3K activation,^1^ was prevented by MKK4 knockdown (Figure 3b). This result is an expected consequence of the interruption of the self-sustaining cycle of ROS → MAP3K → MAP2K → JNK → mitochondrial Sab →↓P-Src → ↑ROS.

As MKK4 has the capacity to activate both JNK and p38, we examined p38 status. Active p38 was identified in cytoplasm under basal conditions and did not increase further after APAP, whereas P-p38 association with mitochondria did increase slightly but not significantly (Figure 3b). Knockdown of MKK4 completely inhibited P-p38 in both cytoplasm and mitochondria (Figure 3b), but we did not observe significant changes in p38 or P-p38 in control mice after APAP treatment. These findings alone do not fully exclude a role for p38 in liver and, therefore, we further addressed the possible contribution of p38 since MKK4 knockdown inhibited basal expression of P-p38 in the liver. These findings with p38 raise the possibility that the protection by MKK4 knockdown, and by inference the prior findings with MLK3 and ASK1 knockout, might be at least partially mediated by p38.

**Figure 4:**
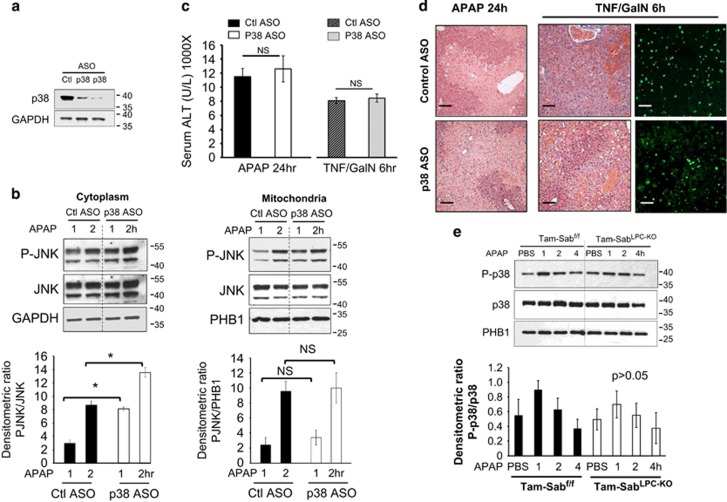
Effect of p38 knockdown in liver injury models. After injection of control or p38 ASO, the mice were treated with APAP (300 mg/kg) or TNF (12 *μ*g/kg)/GalN (800 mg/kg) intraperitoneally. (**a**) Immunoblot analysis of p38 knockdown condition. (**b**) P-JNK, JNK in cytoplasm and translocation to mitochondria in 1 and 2 h after APAP in ASO control *versus* p38 KD mice. Bars represent mean±standard deviation, *n*=5 per group; *P<0.05 (*t*-test), Control *versus* p38 KD. (**c**) Serum ALT 24 h after APAP, 6 h after TNF/GalN. *N*=5 per group; (NS, not significant, ASO control *versus* p38 ASO-treated mice). (**d**) Comparison of hematoxylin and eosin liver histology 24 h after APAP and 6 h after TNF/GalN; TUNEL stain of the latter also shown. Scale bars represent 100 *μ*m. Quantitative microscopy is described in the 'Materials and Methods' section. (**e**) Effect of liver-specific Sab knockout on P-p38 association with mitochondria. Mice were fed with Tam diet for 7 days. One week later, Tam-Sab^f/f^ Control and Tam-Sab^LPC-KO^ KO littermates were treated with APAP (300 mg/kg i.p.) in warm PBS in pyrogen-free PBS. Mitochondria were isolated from livers at indicated time points by differential centrifugation. Western blot analysis was performed using antisera against P-p38, p38 and PHB1. The densitometric ratio of P-p38/PHB1 is shown below the blots (*n*=5); no significant differences were seen by AOV

Although we previously showed that a small molecule p38 inhibitor did not protect, this was done with DMSO to dissolve the inhibitor, which complicates interpretation.^13^ Nakagawa *et al.*^4^ showed that p38*α* haplo-deficient mice were not protected against APAP. However, only 50% loss of p38*α* as well as possible consequences of developmental adaptations might have obscured the importance of p38. Therefore, we assessed the effect of highly efficient p38 knockdown *versus* scrambled control ASO treatment on basal hepatic p38 level and response to APAP or TNF/GalN. p38 ASO decreased expression by >90% (Figure 4a). The response of P-JNK at 1 and 2 h after APAP was not inhibited in cytoplasm or in mitochondrial translocation (Figure 4b). On the contrary, p38 knockdown significantly amplified the increase in sustained P-JNK in cytoplasm at 1 and 2 h after APAP, but did not significantly increase the association of P-JNK with mitochondria. Interestingly, a prior report indicated that liver-specific ablation of p38*α* resulted in excessive JNK activation in the liver after LPS challenge.^14^ Despite the increased P-JNK in p38 knockdown condition, the ALT levels and histological injury in both models were not altered (no protection) with p38 knockdown *versus* scrambled control (Figures 4c and d). A second p38*α*-targeted ASO verified the findings and did not affect the extent of liver injury from APAP or TNF/GalN (Supplementary Figures 1 and 2).

As we did observe P-p38 associated with the mitochondria under basal conditions and some small but not significant increase after APAP, we determined whether Sab was required for this association. Sab is the docking protein of JNK on mitochondria so it seemed possible that it might be responsible for binding of p38. We monitored P-p38 in mitochondrial fraction at 1 to 4 h after APAP in Tam-Sab^LPC-KO^ *versus* Tam-Sab^fl/fl^ littermates. Deletion of Sab did not eliminate the association of P-p38 with mitochondria (Figure 4e), whereas we previously showed that P-JNK was no longer associated.^1^ There was an insignificant transient increase in P-p38 in mitochondrial fraction at 1 h after APAP dosing in both Tam-Sab^f/f^ and Tam-Sab^LPC-KO^ KO mice (Figure 4e). The physiological significance of the constitutive association P-p38 with mitochondria and its activation in the cytoplasm is unknown but does require MKK4.

**Figure 5:**
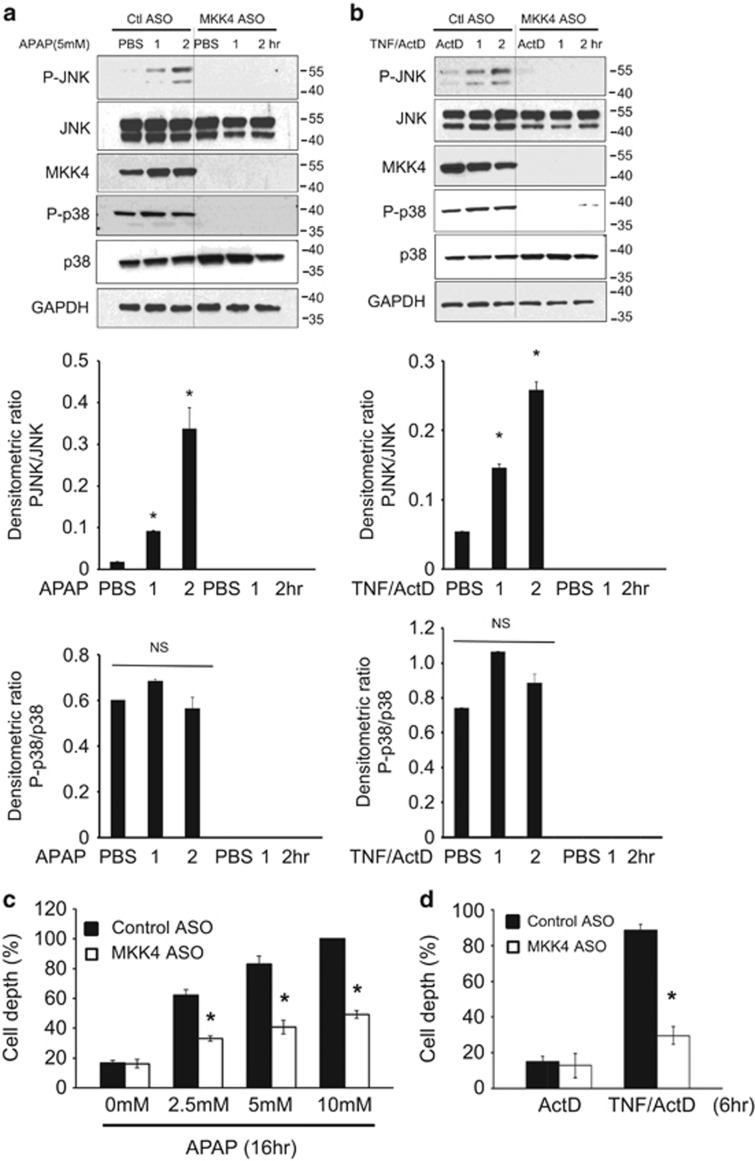
Effect of MKK4 knockdown on APAP and TNF-*α*-induced toxicity in cultured hepatocytes. Immunoblots assessing (**a**) APAP and (**b**) TNF-*α* /ActD-induced JNK activation in PMHs. After control ASO and MKK4 ASO injections, hepatocytes were isolated. Cultured hepatocytes from these mice were incubated with PBS or APAP (5 mM) or TNF-*α* (20 ng/ml)/ActD (0.5 *μ*g/ml), and whole-cell extracts were examined by western blotting for p-JNK and JNK at the indicated times. GAPDH was used as a loading control. Bars represent densitometric analysis of immunoblots from three mice per group. (**c** and **d**) PMHs from control and MKK4 KD mice were incubated with APAP 16 h or (**d**) TNF-*α*/ActD for 6 h, and hepatocytes were stained with Hoechst 3325. Dead cells were counted as described in the 'Materials and Methods' section. *P<0.05 *versus* control group (*n*=three experiments, three different primary culture from three mice)

Finally, to insure that the protection by MKK4 knockdown was not mediated by an effect on nonparenchymal cells and innate immunity/inflammation, we examined the cell autonomous effect of MKK4 knockdown. Hepatocytes were isolated and cultured after *in vivo* treatment with MKK4 or control ASO. The PMH were then exposed to APAP or TNF/actinomycin D for up to 2 h to assess early signaling events. Both toxic treatments activated cellular JNK in controls but prior *in vivo* MKK4 knockdown prevented JNK activation with both treatments (Figures 5a and b). Subsequent cell death was markedly inhibited in both models (Figures 5c and d). Thus, MKK4 in hepatocytes is critical in mediating sustained JNK activation and cell death.

## Discussion

The findings in the current work provide new insights into the mechanisms of hepatotoxicity in two models in which we have previously shown that the interaction of JNK and mitochondrial Sh3bp5 are critical in promoting oxidative stress and sustained JNK activation.^1, 2^ APAP toxicity involves its biotransformation to NAPQI, which directly impairs mitochondrial function, initiating a self-amplifying mitochondrial ROS → MAP3K → MAP2K→ JNK → mitochondrial ROS cycle which ultimately leads to MPT and necrosis.^2, 3, 4, 5, 6, 7, 15, 16^ In the TNF/GalN model, hepatocytes are sensitized to direct TNF-induced apoptosis; galactosamine inhibits antiapoptosis gene expression, particularly genes that dampen TNF-induced mitochondrial ROS production.^17, 18^ As a consequence, JNK leads to Bcl family-mediated mitochondrial outer membrane permeabilization and apoptosis.^8, 9, 19, 20, 21, 22^ In the APAP model, both ASK1 and MLK3 have been shown to participate in activating downstream JNK.^3, 4, 23, 24, 25^ These two MAP3 kinases, activated by ROS, then activate MAP2kinase.^26, 27^ There are two MAP2kinases, which activate JNK: MKK4 and MKK7. MKK4 activates both JNK and p38, whereas MKK7 activates only JNK.^10, 11^ Therefore, we explored the contribution of MKK4 and MKK7 in mouse liver to the activation of JNK and the development of acute liver injury in both the APAP and TNF/GalN models. Furthermore, we examined the role of p38 in APAP toxicity.

We found that efficient ASO knockdown of MKK4, but not ASO knockdown of MKK7, protected against liver injury and sustained JNK activation in both models. As the ASO knockdowns are not hepatocyte specific, we assessed whether the effect of MKK4 knockdown was cell autonomous. PMH cultured after *in vivo* knockdown of MKK4 were protected against APAP or TNF/actinomycin D and JNK activation before PMH cell death was markedly suppressed before the onset of cell death when MKK4 was silenced. To ensure that the protection observed was not due to off-target effects of one specific ASO, all results were confirmed with a second ASO targeting a different portion of the mRNA of MKK4 and MKK7.

Our findings need to be discussed in light of the recent findings of Cubero *et al.*^12^ suggesting that hepatocellular JNK1 and JNK2 dampen hepatotoxicity in liver injury from acute APAP and chronic CCl4. Although we cannot fully explain the apparent discrepancy, these studies involved the combined embryonic liver-specific deletion of JNK1 and global JNK2 deletion and the results were characterized by markedly heightened oxidative stress in response to APAP, which suggested that the embryonic deletions lead to a dampening oxidant defense. A large body of evidence supports the role of JNK in amplifying APAP toxicity, including a variety of approaches such as different small molecule JNK inhibitors,^13, 28, 29^ ASO knockdown of JNK1 and JNK2, JNK2 global knockout,^13, 30^ ASK1 and MLK3 global knockout,^3, 4^ highly specific ASK1 inhibitor,^31^ MKP-1 global knockout,^32^ Sab knockdown and liver-specific inducible knockout of Sab.^1, 2^ The current study adds MKK4 knockdown to the evidence supporting the role of JNK in APAP toxicity while excluding a role for the other downstream substrate of MKK4, namely p38 in mediating toxicity. It appears that the sensitization to APAP toxicity in the combined embryonic JNK1/2 deletion may be accounted for by the observed enhanced mitochondrial oxidant stress observed with this model. However, further work assessing liver-specific inducible deletion of JNK1 and JNK2 may shed more light on this controversy.

We have previously observed by P-MKK4 translocation to mitochondrial with P-JNK.^2^ P-JNK association with mitochondria only occurs in the presence of Sab.^1, 2^ P-MKK4 activation is not sustained in the absence of Sab making it difficult to determine whether P-MKK4 binds directly to Sab or binds to the P-JNK/Sab complex. We have previously shown that P-JNK (plus ATP) directly impairs mitochondrial respiration and increases ROS, which requires binding to Sab.^33, 34^ P-MKK4 plus ATP had no direct effect on mitochondrial function.^1^

As we identified MKK4 as the key MAP2K-mediating JNK activation and toxicity, we addressed the possibility that p38, the other MKK4 known substrate of MKK4, was involved in mediating toxicity. Prior work has suggested that p38 does not contribute to the toxic mechanism of APAP. Gunawan *et al.*^13^ used a p38 inhibitor, which was dissolved in DMSO. Although the study controlled for DMSO vehicle, the APAP injury was markedly dampened by DMSO alone, which therefore complicates the interpretation of finding no additional protection by p38 inhibitor. Nakagawa *et al.*^4^ used p38^+/−^ global hemizygotes and showed no protection against APAP but the decrease in p38 might not have been sufficient to exclude its role. However, in the current study, we used two different ASO targeted treatments to markedly suppress p38 expression in the liver by >90%. Both ASO molecules are specific for the p38*α* isoform and the marked loss of total p38 in immunoblots using antisera to total p38 indicates that nearly all the p38 in liver is p38*α*. The depletion of p38*α* had no effect on the toxicity of APAP or TNF/GalN as reflected in histology and serum ALT. Interestingly, some P-p38 was associated with mitochondria under basal conditions and tended to transiently increase after APAP. This association was the same in Sab knockout liver indicating that Sab is not required for the association of p38 with mitochondria. Furthermore, we previously showed that P-p38 plus ATP had no effect on mitochondrial respiration in contrast to P-JNK plus ATP.^1^ However, we did observe that knockdown of p38 lead to amplification of P-JNK levels in the first few hours after APAP dosing.

A previous study showed that liver-specific p38*α* deletion amplified JNK activation in response to TNF^14^ and a recent report showed that p38 inhibition in cancer cells amplified JNK signaling in response to SMAC mimetic.^35^ These findings might suggest that p38 normally dampens the JNK activation pathway. However, we did not observe an enhancement of the toxicity of APAP or TNF/GalN by depleting p38. Despite the increased P-JNK levels in p38 ASO-treated mice, we did not detect any difference in the association of P-JNK with mitochondria. However, further exploration of the potential protective role of p38 is warranted in future studies, particularly to determine whether p38 knockdown increases the susceptibility to lower nontoxic or minimally toxic doses of APAP and TNF/GalN.

Overall, our findings indicate that MKK4 is the major MAP2K, which activates JNK in acute liver injury. p38, the other downstream target of MKK4, does not promote liver injury from APAP or TNF/galactosamine. Therefore, our findings support the critical role of JNK in hepatotoxicity.

## Materials and methods

### Animals

All animal experiments followed procedures approved by the Institutional Animal Care and Use Committee of the University of Southern California. Male C57BL/6 NHsd mice (6–8 weeks of age) were obtained from ENVIGO Bioproducts Inc. (Madison, WI, USA). *loxP*-flanked Sab mutant mice (Sh3bp5^tm1a(KOMP)Wtsi^) were generated in C57BL/6N background by KOMP, UC Davis. Exon 5, 6 and 7 were flanked by *loxP*. Primers for genotyping of Sab mutant mice are: for floxed mice 5′-GAGATGGCGCAACGCAATTAATG-3′ and 5′-TGTGTAGCAGAGTAGACCCTCATGC-3′, for post-Cre mice 5′-GCTACCATTACCAGTTGGTCTGGTGTC-3′ and 5′-TGTGTAGCAGAGTAGACCCTCATGC-3′. To generate tamoxifen-induced hepatocyte-specific Sab KO mice (Tam-Sab^LPC-KO^), Sab floxed mice were crossbred with Alb-Cre^ERT2^ mice (Alb^tm1(cre/ERT2)Mtz^)^36, 37^ kindly provided by Dr. Pierre CHAMBON, Institut de Génétique et de Biologie Moléculaire et Cellulaire (IGBMC), France. Sab^f/f^;Alb-Cre^ERT2+/-^ were crossbred with Sab^f/f^ floxed mice at least 10 times, and then 6-week-old male mice were fed with tamoxifen (Tam) 1 g/kg of diet pellet for 7 days and the experiment was performed 5–7 days later after the last day of Tam diet.^1^

### Preparation of antisense oligonucleotide (ASO) and *in vivo* knockdown

MKK4 ASO (5′-TACGCTGGCTCTAAGC-3′), and its control ASO (5′-GGCCAATACGCCGTCA-3′); MKK7 ASO (5′-ACTTTGGTCTCTTCCTGTGA-3′), MKK7 ASO (5′-ACTTTGGTCTCTTCCTGTGA-3′), or p38*α* ASO (5′-GCAGCCTCTCTCTGTCACTG-3′) and its control ASO (5′-CCTTCCCTGAAGGTTCCTCC-3′) were provided by Ionis Pharmaceuticals. Experiments with a second set of ASO and control ASO were conducted to verify the findings: MKK4 ASO (5′-GTGGTTTAGTGCTTTC-3′) and its control ASO (5′-GGCCAATACGCCGTCA-3′); MKK7 ASO (5′-CCGTTCACAGTGTCTGTCGG-3′) or p38*α* ASO (5′-CCCGCTCTGAATCCAGATCT-3′) and its control ASO (5′-CCTTCCCTGAAGGTTCCTCC-3′). The results are shown in the supplemental section. ASO was dissolved in sterile PBS and oligonucleotide concentration was determined before aliquot for storage in −80 °C. Eight-week-old mice were injected 50 mg/kg intraperitoneally on alternate days for seven doses. The experiment was performed the next day after last injection.^1, 2, 5^

### *In vivo* hepatotoxicity models

Acetaminophen (APAP, Sigma Aldrich, St Louis, MO, USA #A7085) was dissolved in 50 °C PBS by vigorous vortexing and brought back to 25 °C before injection. Overnight fasted mice received PBS or APAP 300 mg/kg intraperitoneally and blood and samples were collected at 0.5, 1, 2, 4 or 24 h after injection. Galactosamine (GalN, Sigma #G0500) was dissolved in PBS. TNF*α* (Calbiochem-Millipore, Billerica, MA, USA #654245) was dissolved and diluted in PBS. Overnight fasted mice received GalN (800 mg/kg) 30 min before PBS or TNF*α* (12 *μ*g/kg) intraperitoneally. The blood and samples were collected at 1, 2 or 24 h after injection.

### Histology and immunofluorescence staining

The liver sections were fixed in 10% neutral buffer formalin and embedded in paraffin for 5 *μ*m thick section. The tissue sections were stained for hematoxylin and eosin (H&E) and the images were taken with Nikon Eclipse *80i* (Melville, NY, USA). For TUNEL staining, the tissue sections were stained using *In Situ* Cell Death Detection Kit, Fluorescein (Roche, Burlington, NC, USA) and images were taken with Leica SP8 (LAS X) confocal microscope used with HC PL APO CS2 20X/0.75 DRY lens. Laser intensity used was 1% for both DAPI and FITC, gain was 752 to 800 g for DAPI and 726 to 750 g for FITC. (Pinhole 1 AU =56.6 *μ*M, average =4). Exposure was identical in all cases based on one scan of the same duration for each fluorescence imaging.

### Quantitative RT-PCR

Total mRNA was extracted from mouse liver tissue by RNeasy Plus Mini kit (Qiagen, Valencia, CA, USA). cDNA was synthetized by QIAGEN OneStep RT-PCR Kit using 500 ng of mRNA. A total 2.5 ng of cDNA was applied for quantitive PCR in ABI 7900 HT Fast Real-Time PCR System using QuantiTect SYBR Green PCR Kit. Specific primer-amplified RNA amount was determined from standard curve generated by serial dilution of pool mRNA of normal three male mice. MKK4 or MKK7 mRNA was normalized by housekeeping gene TBP (TATA box binding protein). Primers for MKK4 mRNA are 5′-AATCGACAGCACGGTTTACTC-3′ and 5′-TGAAATCCCAGTGTTGTTCAGG-3′. Primers for MKK7 are 5′-GATGTCGCGTCCTGGTTTAAG-3′ and 5′-ACTTGGGAGAAGGTGGGGAA-3′. Primers for TBP are 5′-AGAACAATCCAGACTAGCAGCA-3′ and 5′-GGGAACTTCACATCACAGCTC-3′.

### Isolation of liver mitochondria and cytoplasm

Mitochondria were isolated from mouse liver by differential centrifugation as described before.^1, 2^ Livers were homogenized in homogenizing buffer (250 mM sucrose, 10 mM Tris, 2 mM EGTA, pH 7.4 in ice) and centrifuged at 1000X g for 10 min to collect fibrous tissue debris and nuclei. Mitochondria were collected by centrifugation at 9000 Xg for 10 min. The mitochondrial pellet was washed once. Mitochondrial protein was extracted in RIPA lysis buffer for Western blot analysis. The supernatant (cytoplasmic fraction) and mitochondrial lysate were stored in −80 °C.

### Antibodies and reagents

Antisera to P-JNK (#4668), SEK1/MKK4 (5C10; #3346), Phospho-SEK1/MKK4 (Ser257; C36C11; #4514), P-Src(Tyr416; #6943), c-Src (2109), P-p38 (#4511), total p38 (#8690), MKK7 (#4172), PHB1 (#2426; Cell Signaling Technology, Danvers, MA, USA), total JNK (JNK1/2/3; sc571; Santa Cruz Biotechnology, Dallas, TX, USA), GAPDH (G9295; Sigma-Aldrich) were used. ALT was determined by reagents from Teco Diagnostics (Anaheim, CA, USA). Covalent binding to liver proteins was assessed using antiserum to NAPQI protein adducts provided by Dr. Laura James of University of Arkansas for Medical Sciences. GSH was determined as described before.^38^

### Western blot analysis

Aliquots of cytoplasmic or mitochondrial extracts were fractionated by electrophoresis on 7.5, 10 or 12% SDS-polyacrylamide gel (Bio-Rad, Hercules, CA, USA). Subsequently, proteins were transferred to nitrocellulose membrane using iBlot Gel transfer device (Invitrogen, Grand Island, NY, USA), and blots were blocked with 5% (w/v) nonfat milk or 5% (w/v) BSA dissolved in Tris buffered saline with 0.05% Tween 20 (TBS-T). The blots were then incubated with the desired primary and secondary antibodies. Finally, the proteins were detected by ECL reagent (Thermo Scientific or GE Lifesciences, Waltham, MA, USA) using autoradiography film (Denville) or Amersham Hyperfilm ECL (GE Lifesciences). All gels shown are representative samples from at least three experiments.

### Cell isolation, culture and cell death assay

Primary mouse hepatocytes (PMHs) from control or MKK4 ASO-injected mice were isolated and cultured as described previously.^1, 39^ Three hours after plating of isolated hepatocytes, APAP (5 mM) dissolved in fresh prewarmed DMEM/F-12 culture medium was added. After 16 h of treatment, the cells were double-stained with Hoechst 33258 (8 *μ*g/ml; Invitrogen) and SYTOX Green (1 *μ*mol/l; Invitrogen). In other experiments, hepatocytes from control or MKK4 ASO-injected mice were incubated with TNF-*α* (20 ng/ml) /actinomycin D (ActD; 0.5 *μ*g/ml) and, after 6 h, stained with Hoechst 33258 and apoptotic cells were counted. Quantification of total and apoptotic cells was performed by counting a minimum of 1000 cells in 10 different fields. Necrotic cells (SYTOX green-positive) were determined by counting the same field under Nikon Eclipse TE300 fluorescence microscope using MetaMorph imaging program.^33, 34^

### Statistics

A minimum of three biological replicates were considered for all the studies. The data are expressed as mean±S.D. Statistical analyses were performed using the paired two-tailed Student’s *t*-test and analysis of variance. *P*-value <0.05 was defined as statistically significant.

## References

[bib1] Win S, Than TA, Min RW, Aghajan M, Kaplowitz N. c-Jun N-terminal kinase mediates mouse liver injury through a novel Sab (SH3BP5)-dependent pathway leading to inactivation of intramitochondrial Src. Hepatology 2016; 63: 1987–2003. 26845758 10.1002/hep.28486 PMC4874901

[bib2] Win S, Than TA, Han D, Petrovic LM, Kaplowitz N. C-Jun N-terminal kinase (JNK)-dependent acute liver injury from acetaminophen or tumor necrosis factor (TNF) requires mitochondrial Sab protein expression in mice. J Biol Chem 2011; 286: 35071–35078. 21844199 10.1074/jbc.M111.276089 PMC3186406

[bib3] Sharma M, Gadang V, Jaeschke A. Critical role for mixed-lineage kinase 3 in acetaminophen-induced hepatotoxicity. Mol Pharmacol 2012; 82: 1001–1007. 22918968 10.1124/mol.112.079863 PMC3477232

[bib4] Nakagawa H, Maeda S, Hikiba Y, Ohmae T, Shibata W, Yanai A et al. Deletion of apoptosis signal-regulating kinase 1 attenuates acetaminophen-induced liver injury by inhibiting c-Jun N-terminal kinase activation. Gastroenterology 2008; 135: 1311–1321. 18700144 10.1053/j.gastro.2008.07.006

[bib5] Hanawa N, Shinohara M, Saberi B, Gaarde WA, Han D, Kaplowitz N. Role of JNK translocation to mitochondria leading to inhibition of mitochondria bioenergetics in acetaminophen-induced liver injury. J Biol Chem 2008; 283: 13565–13577. 18337250 10.1074/jbc.M708916200 PMC2376214

[bib6] Kon K, Kim JS, Jaeschke H, Lemasters JJ. Mitochondrial permeability transition in acetaminophen-induced necrosis and apoptosis of cultured mouse hepatocytes. Hepatology 2004; 40: 1170–1179. 15486922 10.1002/hep.20437

[bib7] Saito C, Lemasters JJ, Jaeschke H. c-Jun N-terminal kinase modulates oxidant stress and peroxynitrite formation independent of inducible nitric oxide synthase in acetaminophen hepatotoxicity. Toxicol Appl Pharmacol 2010; 246: 8–17. 20423716 10.1016/j.taap.2010.04.015 PMC2885557

[bib8] Liu J, Lin A. Role of JNK activation in apoptosis: a double edged sword. Cell Res 2005; 15: 36–42. 15686625 10.1038/sj.cr.7290262

[bib9] Tsuruta F, Sunayama J, Mori Y, Hattori S, Shimizu S, Tsujimoto Y et al. JNK promotes Bax translocation to mitochondria through phosphorylation of 14-3-3 proteins. EMBO J 2004; 23: 1889–1899. 15071501 10.1038/sj.emboj.7600194 PMC394248

[bib10] Whitmarsh AJ, Davis RJ. Role of mitogen-activated protein kinase kinase 4 in cancer. Oncogene 2007; 26: 3172–3184. 17496914 10.1038/sj.onc.1210410

[bib11] Tournier C, Dong C, Turner TK, Jones SN, Flavell RA, Davis RJ. MKK7 is an essential component of the JNK signal transduction pathway activated by proinflammatory cytokines. Genes Dev 2001; 15: 1419–1426. 11390361 10.1101/gad.888501 PMC312702

[bib12] Cubero FJ, Zoubek ME, Hu W, Peng J, Zhao G, Nevzorova YA et al. Combined Activities of JNK1 and JNK2 in Hepatocytes Protect Against Toxic Liver Injury. Gastroenterology 2016; 150: 968–981. 26708719 10.1053/j.gastro.2015.12.019 PMC5285516

[bib13] Gunawan BK, Liu ZX, Han D, Hanawa N, Gaarde WA, Kaplowitz N. c-Jun N-terminal kinase plays a major role in murine acetaminophen hepatotoxicity. Gastroenterology 2006; 131: 165–178. 16831600 10.1053/j.gastro.2006.03.045

[bib14] Heinrichsdorff J, Luedde T, Perdiguero E, Nebreda AR, Pasparakis M. p38 alpha MAPK inhibits JNK activation and collaborates with IkappaB kinase 2 to prevent endotoxin-induced liver failure. EMBO Rep 2008; 9: 1048–1054. 18704119 10.1038/embor.2008.149 PMC2572111

[bib15] Chen W, Koenigs LL, Thompson SJ, Peter RM, Rettie AE, Trager WF et al. Oxidation of acetaminophen to its toxic quinone imine and nontoxic catechol metabolites by baculovirus-expressed and purified human cytochromes P450 2E1 and 2A6. Chem Res Toxicol 1998; 11: 295–301. 9548799 10.1021/tx9701687

[bib16] Han D, Dara L, Win S, Than TA, Yuan L, Abbasi SQ et al. Regulation of drug-induced liver injury by signal transduction pathways: critical role of mitochondria. Trends Pharmacol Sci 2013; 34: 243–253. 23453390 10.1016/j.tips.2013.01.009 PMC3622802

[bib17] Hoffmann F, Sass G, Zillies J, Zahler S, Tiegs G, Hartkorn A et al. A novel technique for selective NF-kappaB inhibition in Kupffer cells: contrary effects in fulminant hepatitis and ischaemia-reperfusion. Gut 2009; 58: 1670–1678. 19470497 10.1136/gut.2008.165647

[bib18] Kamata H, Honda S, Maeda S, Chang L, Hirata H, Karin M. Reactive oxygen species promote TNFalpha-induced death and sustained JNK activation by inhibiting MAP kinase phosphatases. Cell 2005; 120: 649–661. 15766528 10.1016/j.cell.2004.12.041

[bib19] Basu A, Haldar S. Identification of a novel Bcl-xL phosphorylation site regulating the sensitivity of taxol- or 2-methoxyestradiol-induced apoptosis. FEBS Lett 2003; 538: 41–47. 12633850 10.1016/s0014-5793(03)00131-5

[bib20] Morel C, Carlson SM, White FM, Davis RJ. Mcl-1 integrates the opposing actions of signaling pathways that mediate survival and apoptosis. Mol Cell Biol 2009; 29: 3845–3852. 19433446 10.1128/MCB.00279-09 PMC2704749

[bib21] Cazanave SC, Gores GJ. The liver's dance with death: two Bcl-2 guardian proteins from the abyss. Hepatology 2009; 50: 1009–1013. 19787811 10.1002/hep.23188 PMC2906400

[bib22] Masuoka HC, Mott J, Bronk SF, Werneburg NW, Akazawa Y, Kaufmann SH et al. Mcl-1 degradation during hepatocyte lipoapoptosis. J Biol Chem 2009; 284: 30039–30048. 19734538 10.1074/jbc.M109.039545 PMC2781558

[bib23] Xie Y, Ramachandran A, Breckenridge DG, Liles JT, Lebofsky M, Farhood A et al. Inhibitor of apoptosis signal-regulating kinase 1 protects against acetaminophen-induced liver injury. Toxicol Appl Pharmacol 2015; 286: 1–9. 25818599 10.1016/j.taap.2015.03.019 PMC4444402

[bib24] Patterson AD, Carlson BA, Li F, Bonzo JA, Yoo MH, Krausz KW et al. Disruption of thioredoxin reductase 1 protects mice from acute acetaminophen-induced hepatotoxicity through enhanced NRF2 activity. Chem Res Toxicol 2013; 26: 1088–1096. 23697945 10.1021/tx4001013 PMC6334300

[bib25] Nishitoh H, Matsuzawa A, Tobiume K, Saegusa K, Takeda K, Inoue K et al. ASK1 is essential for endoplasmic reticulum stress-induced neuronal cell death triggered by expanded polyglutamine repeats. Genes Dev 2002; 16: 1345–1355. 12050113 10.1101/gad.992302 PMC186318

[bib26] Jaeschke A, Davis RJ. Metabolic stress signaling mediated by mixed-lineage kinases. Mol Cell 2007; 27: 498–508. 17679097 10.1016/j.molcel.2007.07.008 PMC1986670

[bib27] Brancho D, Ventura JJ, Jaeschke A, Doran B, Flavell RA, Davis RJ. Role of MLK3 in the regulation of mitogen-activated protein kinase signaling cascades. Mol Cell Biol 2005; 25: 3670–3681. 15831472 10.1128/MCB.25.9.3670-3681.2005 PMC1084312

[bib28] Latchoumycandane C, Goh CW, Ong MM, Boelsterli UA. Mitochondrial protection by the JNK inhibitor leflunomide rescues mice from acetaminophen-induced liver injury. Hepatology 2007; 45: 412–421. 17366662 10.1002/hep.21475

[bib29] Henderson NC, Pollock KJ, Frew J, Mackinnon AC, Flavell RA, Davis RJ et al. Critical role of c-jun (NH2) terminal kinase in paracetamol- induced acute liver failure. Gut 2007; 56: 982–990. 17185352 10.1136/gut.2006.104372 PMC1994347

[bib30] Bourdi M, Korrapati MC, Chakraborty M, Yee SB, Pohl LR. Protective role of c-Jun N-terminal kinase 2 in acetaminophen-induced liver injury. Biochem Biophys Res Commun 2008; 374: 6–10. 18586006 10.1016/j.bbrc.2008.06.065 PMC2574690

[bib31] Xie Y, Ramachandran A, Breckenridge DG, Liles JT, Lebofsky M, Farhood A et al. Inhibitor of apoptosis signal-regulating kinase 1 protects against acetaminophen-induced liver injury. Toxicol Appl Pharmacol 2015; 286: 1–9. 25818599 10.1016/j.taap.2015.03.019 PMC4444402

[bib32] Wancket LM, Meng X, Rogers LK, Liu Y. Mitogen-activated protein kinase phosphatase (Mkp)-1 protects mice against acetaminophen-induced hepatic injury. Toxicol Pathol 2012; 40: 1095–1105. 22623522 10.1177/0192623312447551 PMC3504636

[bib33] Win S, Than TA, Fernandez-Checa JC, Kaplowitz N. JNK interaction with Sab mediates ER stress induced inhibition of mitochondrial respiration and cell death. Cell Death Dis 2014; 5: e989. 24407242 10.1038/cddis.2013.522 PMC4040675

[bib34] Win S, Than TA, Le BH, García-Ruiz C, Fernandez-Checa JC, Kaplowitz N. Sab (Sh3bp5) dependence of JNK mediated inhibition of mitochondrial respiration in palmitic acid induced hepatocyte lipotoxicity. J Hepatol 2015; 62: 1367–1374. 25666017 10.1016/j.jhep.2015.01.032 PMC4439305

[bib35] Lalaoui N, Hänggi K, Brumatti G, Chau D, Nguyen NY, Vasilikos L et al. Targeting p38 or MK2 Enhances the Anti-Leukemic Activity of Smac-Mimetics. Cancer Cell 2016; 29: 145–158. 26859455 10.1016/j.ccell.2016.01.006

[bib36] Metzger D, Clifford J, Chiba H, Chambon P. Conditional site-specific recombination in mammalian cells using a ligand dependent chimeric Cre recombinase. Proc Natl Acad Sci USA 1995; 92: 6991–6995. 7624356 10.1073/pnas.92.15.6991 PMC41457

[bib37] Feil R, Brocard J, Mascrez B, LeMeur M, Metzger D, Chambon P. Ligand-activated site-specific recombination in mice. Proc Natl Acad Sci USA 1996; 93: 10887–10890. 8855277 10.1073/pnas.93.20.10887 PMC38252

[bib38] Rahman I, Kode A, Biswas SK. Assay for quantitative determination of glutathione and glutathione disulfide levels using enzymatic recycling method. Nat Protoc 2006; 1: 3159–3165. 17406579 10.1038/nprot.2006.378

[bib39] Han D, Hanawa N, Saberi B, Kaplowitz N. Hydrogen peroxide and redox modulation sensitize primary mouse hepatocytes to TNF-induced apoptosis. Free Radic Biol Med 2006; 41: 627–639. 16863996 10.1016/j.freeradbiomed.2006.05.002

