# Supplementary material for: The role of MAP2 kinases and p38 kinase in acute murine liver injury models
Source: Cell Death Dis. 2017 Jun 29;8(6):e2903–. doi: 10.1038/cddis.2017.295 (PMC5584575; doi:10.1038/cddis.2017.295)
Supplement: Supplementary Figure 1 and 2 [file cddis2017295x1.pdf]

Supplementary figure 1

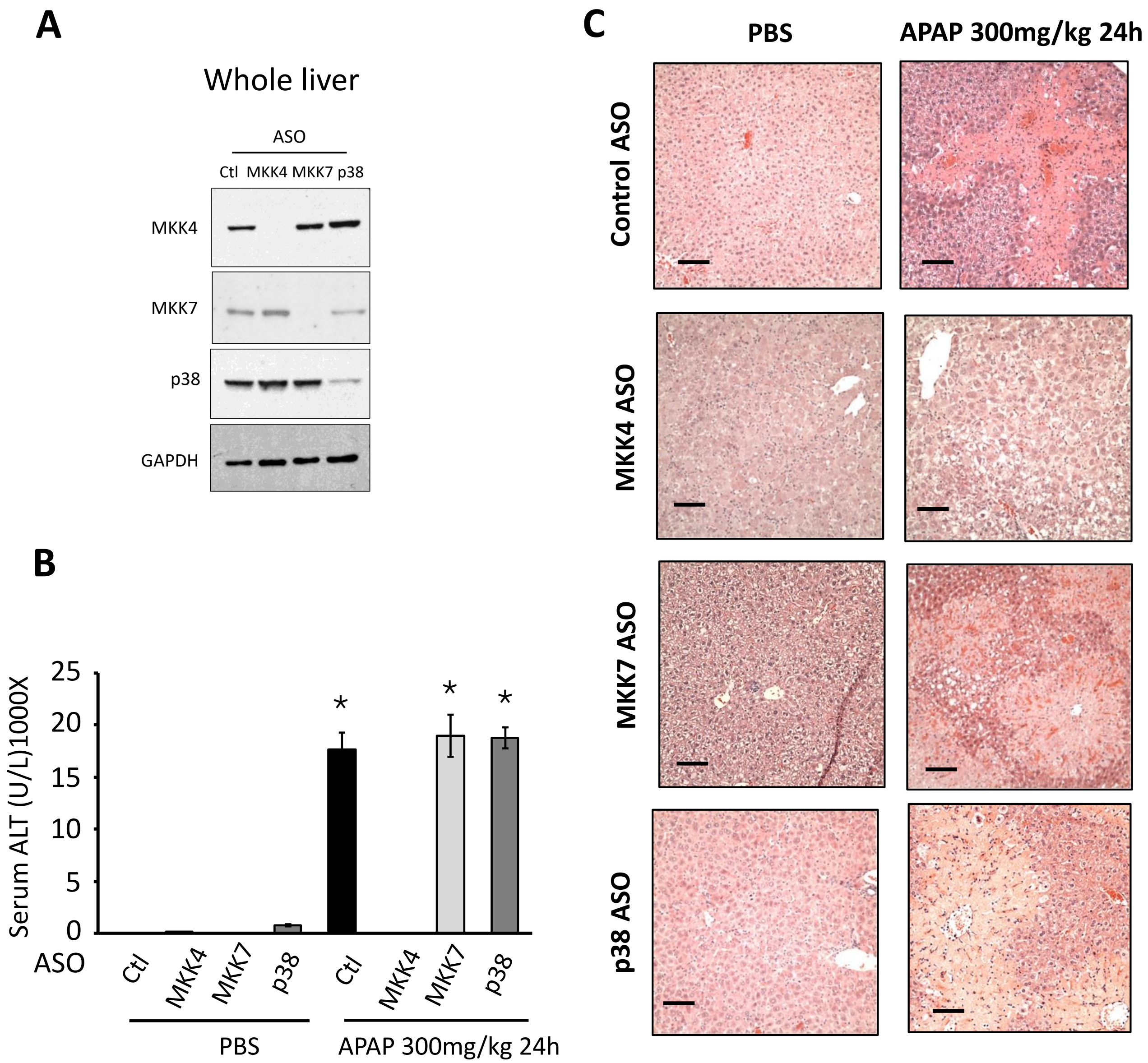

Supplementary Figure.1. Effect of control ASO, MKK4 ASO,MKK7 ASO, p38 ASO knockdown in liver injury models using different ASO than in Results. After injection of control or MKK4, MKK7, p38 ASO, mice were treated with APAP (300 mg/kg) intraperitoneally. . (**A**) Western blots of MKK4, MKK7 and p38 knockdown in liver lysate. Basal level and efficiency of knockdown of MKK4 ASO, MKK7 ASO and p38 ASO. (**B**) serum ALT (n = 5 per group). ✱= p<0.05 versus correspondingly PBS control. (**C**) H & E stain. Scale bars represent 100µm.

Supplementary figure 2

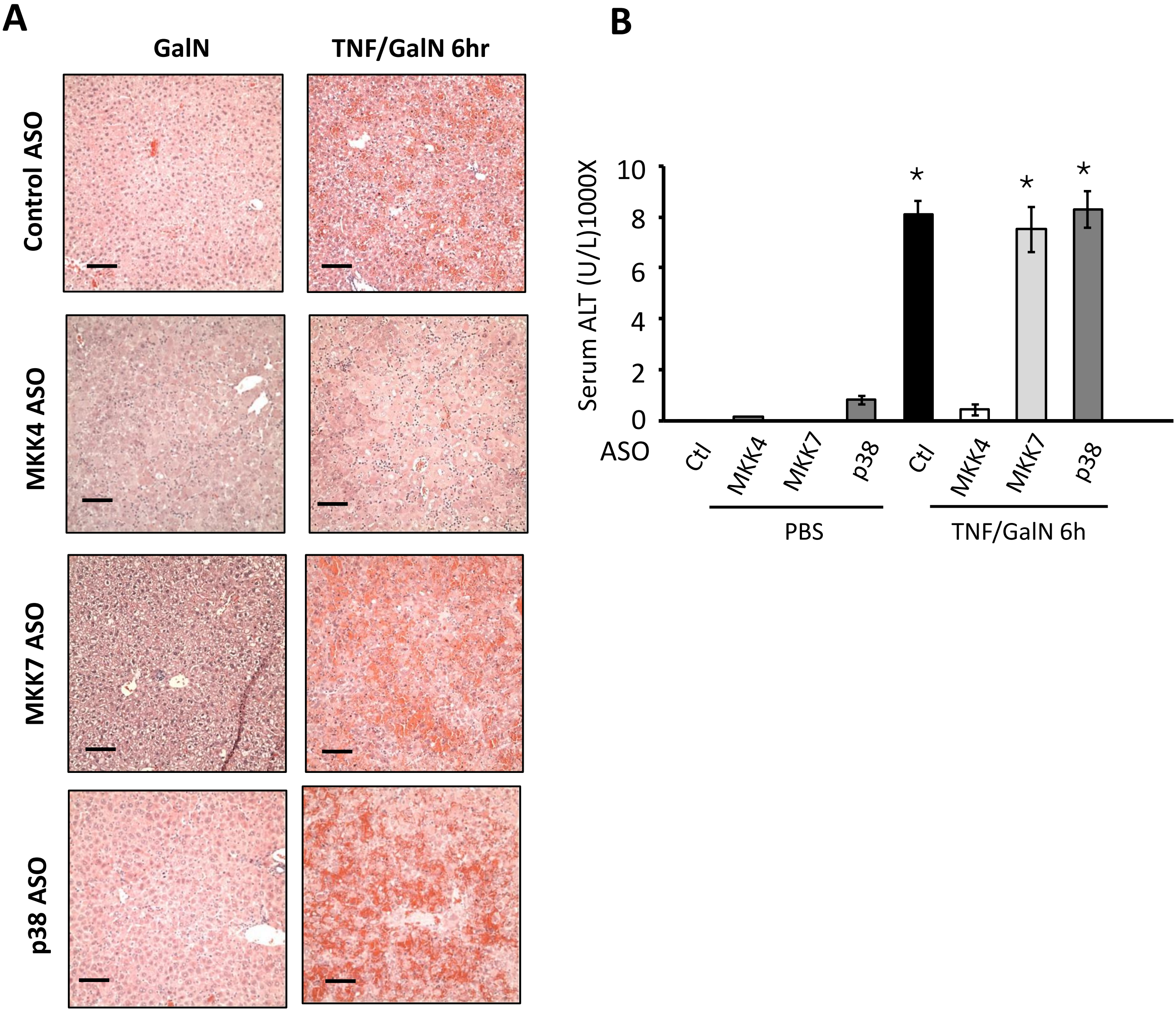

Supplementary Figure.2. Effect of control ASO, MKK4 ASO ,MKK7 ASO, p38 ASO knockdown in liver injury models using different ASO than in Results. After injection of control or MKK4, MKK7, p38 ASO (2), mice were treated with TNF (12 μg/kg)/GalN (800 mg/kg) intraperitoneally for 6 hours. (**A**) Representative H & E stain (**B**) serum ALT. Scale bars represent 100μm. Representative of 5 different mice. (n = 5 per group) . *****= $p<0.05$ versus PBS control.
